# Predicting hypertension using machine learning: Findings from Qatar Biobank Study

**DOI:** 10.1371/journal.pone.0240370

**Published:** 2020-10-16

**Authors:** Latifa A. AlKaabi, Lina S. Ahmed, Maryam F. Al Attiyah, Manar E. Abdel-Rahman

**Affiliations:** Department of Public Health, College of Health Science, QU Health, Qatar University, Doha, Qatar; International University of Health and Welfare, School of Medicine, JAPAN

## Abstract

**Background and objective:**

Hypertension, a global burden, is associated with several risk factors and can be treated by lifestyle modifications and medications. Prediction and early diagnosis is important to prevent related health complications. The objective is to construct and compare predictive models to identify individuals at high risk of developing hypertension without the need of invasive clinical procedures.

**Methods:**

This is a cross-sectional study using 987 records of Qataris and long-term residents aged 18_+_ years from Qatar Biobank. Percentages were used to summarize data and chi-square tests to assess associations. Predictive models of hypertension were constructed and compared using three supervised machine learning algorithms: decision tree, random forest, and logistics regression using 5-fold cross-validation. The performance of algorithms was assessed using accuracy, positive predictive value (PPV), sensitivity, F-measure, and area under the receiver operating characteristic curve (AUC). Stata and Weka were used for analysis.

**Results:**

Age, gender, education level, employment, tobacco use, physical activity, adequate consumption of fruits and vegetables, abdominal obesity, history of diabetes, history of high cholesterol, and mother’s history high blood pressure were important predictors of hypertension. All algorithms showed more or less similar performances: Random forest (accuracy = 82.1%, PPV = 81.4%, sensitivity = 82.1%), logistic regression (accuracy = 81.1%, PPV = 80.1%, sensitivity = 81.1%) and decision tree (accuracy = 82.1%, PPV = 81.2%, sensitivity = 82.1%. In terms of AUC, compared to logistic regression, while random forest performed similarly, decision tree had a significantly lower discrimination ability (p-value<0.05) with AUC’s equal to 85.0, 86.9, and 79.9, respectively.

**Conclusions:**

Machine learning provides the chance of having a rapid predictive model using non-invasive predictors to screen for hypertension. Future research should consider improving the predictive accuracy of models in larger general populations, including more important predictors and using a variety of algorithms.

## Introduction

Hypertension is a common health condition that has become an issue in the modern world; it is part of the metabolic syndrome and a multifactorial condition in which an individual is diagnosed with systolic blood pressure ≥140 mmHg and/ or a diastolic pressure ≥90 mmHg. Its exact causes are unknown but genetic mutation, increased sodium intake, decreased physical activity, and obesity contribute to its progression [1]. In some cases, hypertension acts as a “silent killer;” only noticed when it reaches a dangerous level [2].

According to the World Health Organization (WHO), hypertension globally contributes to 12.8% of the total deaths, and approximately causes 7.5 million deaths [3]. The STEPwise survey conducted in Qatar in 2012 showed that 32.9% of the population aged 18–64 years were hypertensive or on medication for hypertension; 28.0% of females and 37.7% males, while 31.4% were not on medication for hypertension [4].

For some people, hypertension treatment may include only lifestyle adjustments without the use of medications. Intervening into lifestyle includes, but not limited to, reducing salt intake, adopting a low-fat diet, consuming more fruits and vegetables, adopting an active lifestyle, and quitting smoking [1]. One successful prevention approach is to specify those at high risk and target them. Research has shown that the development of hypertension is not only influenced by prehypertension status, but also by other factors such as age [5], gender [6], diet [7], body mass index [8], literacy level [9], stress [10], comorbidities [11] as well as clinical parameters [12–15].

Due to the huge costs of chronic diseases, studies were conducted to estimate the risk of hypertension, to prevent further costly management and treatment of complications. Conventional logistic regression was applied in many of these studies.

Predictive models are useful in predicting hypertension, being essential in medical practice because of their value in patients’ care [16]. Used in clinical settings, the Framingham hypertension risk score, a gender-specific algorithm, is used to predict the risk of developing cardiovascular diseases in 10-year time [17]. It is one of the main scores used to indicate hypertension. Many methods utilizing machine learning (ML) techniques are used in risk models of hypertension, e.g., artificial neural network, support vector machine, random forest, naive bayes classifier, gradient boosting machines, decision tree, and logistic regression [18–20]. Echouffo-Tcheugui et al. systematical reviewed the performance of such algorithms [20], and Krittanawong et al. gave a comprehensive review on the prediction of hypertension using artificial intelligence [19].

Using several ML techniques, a number of predictive factors were identified to predict hypertension, e.g., comorbidity, medication history, age >60 years, sex, smoking, family history of hypertension, body mass index, educational level, salty diet, vegetable, fruit and meat consumption, regular physical exercise, low-density lipids, occupational status, depression and anxiety status [21–24].

According to the American Heart Association, hypertension, obesity and ageing are the leading risk factors to cardiovascular diseases, costing the USA’s health system, around $555 billion in 2016, which is estimated to double to $1.1 trillion in 2035 [25]. On the other hand, the costs per episode of care for hypertension in low and middle-income countries ranges between $500 and $1500 or monthly costs of treatment around $22 [26]. Additionally, people often develop hypertension in their middle age, which leads to a loss in productive years, incurring the healthcare system more expenses [27]. Limited data is available on the economic burden of hypertension in Qatar. Early identification of hypertensive patients will contribute to reducing the cost and economic burdens of hypertension on any healthcare system. These individuals at high risk may be identified using predictive models based on baseline data easily obtained from non-intrusive procedures. Such individuals may be sent to health facilities for preventive measures. Besides serving as a pilot for future studies, the contribution of this study to human knowledge is important in terms of applying new methods to diagnose individuals with hypertension easily and, most importantly, without unnecessary extra expenditures or resources that can be utilized elsewhere.

The objective of this study was to construct and compare logistic regression predictive models and decision tree techniques, using ML, to identify individuals at high risk of developing hypertension without the need of invasive clinical procedures.

## Methods

### Study design and data sources

This is a cross-sectional study using data from 1,000 Qatari and long-term residents (residing in Qatar for 15 years or more) aged 18 years or older from the Qatar Biobank (QBB) study [28]. Exclusion criteria included pregnant women whose results of hypertension may vary during stages of pregnancy [29]. In summary, the QBB study is a population-based longitudinal cohort database that collects data and biological samples. Voluntary enrolment in this study started in 2012, and recruitment reached 18,000 in 2019 of relatively young, mostly Qataris, highly educated and affluent population, thus not representing the general population of Qatar [30, 31]. Our study sample size was randomly selected by QBB from this population. In addition to biological samples, self-administered questionnaire interviews on individuals’ lifestyles, current and past medical and family history, anthropometrics, spirometry, and blood pressure measurement were collected [32].

### Outcome variable

The outcome variable was hypertension; a binary variable identifying participants as hypertensive or normotensive. As per the WHO’s definition, a participant was considered hypertensive if he/she has a systolic blood pressure ≥140 mmHg and/ or a diastolic pressure ≥90 mmHg or is taking hypertension medication. The blood pressure measurements, obtained in our study sample, were the average of repeated measurements taken by QBB nurses from each participant using an Omron 705 automated device in a sitting position. Two diastolic and systolic blood pressure measurements were collected within five-minute intervals, and if these two measurements differed by 5 mmHg or more, a third measurement was taken to ensure the reliability of results [28]. Although blood pressure was not measured in repeated visits as recommended, QBB trained nurses used standardized procedures to control for measurement error.

### Predictors

Predictors’ selection was guided by the literature and their availability in the QBB study. Sociodemographic variables included age categorized into <50 or 50+ years, gender, education level (primary or below, secondary or higher), and employment (yes or no). Lifestyle variables included physical activity, tobacco use, and frequency of consuming adequate fruits or vegetables.

Studies showed that self-reported physical activity is inversely associated with the development of hypertension [33]. Low physical activity is a major issue in Qatar, present in about 46% of the population [34]. In this study, a participant was considered active if he/she had 150 minutes or more of exercise during a typical week. Tobacco use was defined as using smoked products and/ or water pipes (Shisha). Using age at first use, participants who reported using tobacco in the same year of data collection were considered as none users; there were only eight such participants. In Qatar, about one in five men were smokers in 2013, indicating a significant health problem in the country [35]. Thus, it is an important variable to consider in this study as it is known to be associated with several complications leading to elevated blood pressure [36]. The Dietary Approaches to Stop Hypertension (DASH) includes a diet rich in fruits and vegetables, together with low-fat dairy products, fiber, and minerals. Participants in this study were considered consuming adequate fruits or vegetables if they had 4 or more servings each of fresh fruit and vegetable items as collected by the QBB diet questionnaire [28].

In Qatar, abdominal obesity is present among 40% and 45% of females and males [37]. Obesity is an important health problem in Qatar; it was found to be associated with hypertension, diabetes, and lifestyle factors [38, 39]. Based on waist circumference, abdominal obesity was categorized in this study using Qatari specific cutoff values of ≥102 cm for men and ≥94 cm for women [37]. Moreover, being one of the most prevalent diseases in Qatar and globally and ranking as the third lead cause of death, diabetes poses a serious health concern to the Qatari adult population [4, 40, 41]. As an important predictor of hypertension, diabetes was included in this study [23, 42]. Other included comorbidities were the participant’s history of cholesterol and the participant’s mother’s history of hypertension.

### Statistical analysis

The original data was first processed using Stata/MP version 16.1 [43]; new variables were derived were appropriate. All variables utilized in this study were categorical, and thus, percentages were used for summarization and chi-square tests to assess associations. Using the preprocessed data, three prediction models of hypertension were constructed in Weka version 3.8.4 software using three supervised ML algorithms: decision tree (*J48* in Weka), random forest (*RandomTree* in Weka), and logistic regression (*SimpleLogistic* in Weka).

A decision tree, as a classification method, is more commonly used in medical diagnostic protocols because it tests and corresponds to the input data and classifies them into a tree-like structure, which makes them easy to learn and understand. Exploring decision trees in this study is appealing as it can provide adequate visual information to predict if an individual is hypertensive or not. As an algorithm, it creates criteria that successively split data according to values of predictors forming the tree with roots and leaves. Information gain was utilized by the Weka software in splitting criteria from the root node into end-nodes using the selected predictors [44, 45]. Random forest considers the outputs of multiple decision trees, which addresses their sensitivity to the training data resulting in reduction in the variance within the outcome of the data [46]. It is a forest obtained by aggregating many decision trees constructed using subsets of predictors and data records selected randomly and used to rank the importance of variables in a regression or classification problem, where each sample is classified by each tree, and the most common outcome is used as the final classification [47]. The statistical algorithm logistic regression, a popular method in clinical research, was used to predict the probability of each participant of having an event (i.e. being hypertensive) where it models the log odds of this probability as a function of predictor variables [48].

Supervised ML was preferred over other methods because it served the purpose of this research as a classification problem. Supervised ML clarifies the nonlinearity in the data and generates a function mapping input (predictor variables) to output (Hypertension). On the contrary, unsupervised learning models the underlying structure or distribution in the data to learn more about the data. The results produced by supervised ML are more accurate as input data are analyzed, and the predictions are more probably within an acceptable range [45].

Five-fold stratified internal cross-validation was used to evaluate the three algorithms. This method randomly divides the dataset into five equal subsets ensuring that each dataset has the same proportion of hypertensive individuals. Each subset was used by Weka software in turn as a testing dataset; the remaining data was used for training each of the algorithms. Predictions using the training data were evaluated using performance scores, against those using the testing dataset. Five sets of performance measures were computed and averaged. Since the data in this study was limited, five-folds were used in order to get larger samples to training the models, which are statistically representative for the entire dataset and that the model performance scores are not too optimistic or have a broad range of variation [45].

Participants were classified as having a high risk of hypertension if their predicted probability for each person had a cutoff value of 0.5 or more. The performance of algorithms was assessed using the following measures:

$$accuracy\;=\;\frac{TP+TN}{TP+TN+FP+FN}$$ (1)

$$PPV\;=\;\frac{TP}{TP+FN}$$ (2)

$$sensitivity\;=\;\frac{TP}{TP+FP}$$ (3)

$$F-measure\;=\;\frac{2}{\frac{1}{PPV}}+(\frac{1}{sensitivity})$$ (4)

where TP are true-positive, TN is true-negative, FP is false-positive and FN is false-negative. The area under the receiver operating characteristic curve (AUC) was used to evaluate the predictive accuracy of algorithms [49]. AUC measures the discrimination ability of the algorithm in predicting hypertensive and normotensive individuals. It is the area under the curve of plotting TP rates versus FP rates using different cutoff values. The paird ttest (*PairedCorrectedTTester in Weka experimenter)* was used to test for differences between AUC’s after setting a 0.05 as the level of significance.

The importance of variables was ranked using information gain (*InfoGainAttributeEval in Weka*) [45]. Since the outcome variable was unbalanced, with only 14% of the participants being hypertensive, data was preprocessed and augmented using Synthetic Minority Over-sampling Technique (*SMOTE filter in Weka*) [50]. Weka version 3.8.4 default hyperparameters were used to configure all algorithms and the SMOTE filter.

### Ethics statement

The Institutional Review Board (IRB) in QBB has approved the provision of data for this study with a compliment to the ethical principles with an IRB protocol number: Ex -2018-RES-ACC-0119-0061. Ethical approval for this study was obtained from the Qatar University Institutional Review Board with approval number QU-IRB 1256-E/20.

## Results

### Participants characteristics

The study included 987 participants; 13 participants were missing information on hypertension and were excluded. The study sample included 141 participants with hypertension (14.3%) (Table 1). Age was significantly associated with hypertension (p-value<0.001)—about 65% of hypertensive, aged more than 50 years, compared to only 18.4% normotensive. More participants had primary or lower education among hypertension than normotensive (30.5% vs. 10.9%, p-value<0.001). Compared to normotensive, unemployment (47.5% vs. 30.0%, p-value<0.001) and abdominal obesity (50.4% vs. 23.8%, p-value<0.001) were more prevalent among hypertensive participants. Hypertension was more prevalent in participants with a history of diabetes (41.8% vs. 11.2%, p-value<0.001) and history of high cholesterol (60.3% vs. 21.9%, p-value<0.001). Additionally, participants whose mothers had a history of high blood pressure were more vulnerable to be hypertension than normotensive (51.8% vs. 41.4%, p-value = 0.02).

**Table 1:** Baseline characteristics of study participants according to hypertension (n = 987).

Variables	Hypertensive	Normotensive	P-value
**Total sample**	141 (14.3)	846 (85.7)	
**Nationality**			0.845
Qatari	114 (80.9)	687 (80.1)	
Non-Qatari	27 (19.2)	168 (19.9)	
**Age in years**			<0.001
<50	46 (32.6)	690 (81.6)	
50+	95 (67.4)	156 (18.4)	
**Gender**			0.100
Female	62 (44.0)	435 (51.4)	
Male	79 (56.0)	411 (48.6)	
**Education level**			<0.001
Primary or below	43 (30.5)	92 (10.9)	
Secondary+	98 (69.5)	754 (89.1)	
**Employment**			<0.001
No	67 (47.5)	279 (33.0)	
Yes	74 (52.5)	567 (67.0)	
Tobacco use*			0.640
No	104 (73.8)	608 (71.9)	
Yes	37 (26.2)	238 (28.1)	
**Physically active**			0.040
No	82 (58.2)	413 (48.8)	
Yes, 150+ min	59 (41.8)	433 (51.2)	
Fruits & vegetables**			0.590
No	69 (48.9)	435 (51.4)	
Yes	72 (51.1)	411 (48.6)	
**Waist circumference**			<0.001
Normal	70 (49.6)	645 (76.2)	
Higher	71 (50.4)	201 (23.8)	
**History of diabetes**			<0.000
No	82 (58.2)	751 (88.8)	
Yes	59 (41.8)	95 (11.2)	
**History of high cholesterol**			<0.001
No	56 (39.7)	661 (78.1)	
Yes	85 (60.3)	185 (21.9)	
**Mother history of high blood pressure**			0.020
No	68 (48.2)	496 (58.6)	
Yes	73 (51.8)	350 (41.4)	

Data are displayed as n and %.

* Includes smoking and/ or water pipe (Shisha)

** Adequate consumption of fruits or vegetables (4 or more servings each of fresh fruit and vegetable items listed in the QBB questionnaire)

### Predictors of hypertension

ML was employed to examine the performance of three algorithms (decision tree, random forest, logistic regression) using eleven variables of which seven were non-clinical and non-invasive variables (age, sex, education, employment, tobacco use, physical activity and adequate consumption of fruits and vegetables), and four were easily obtainable clinical variables (mother history of hypertension, history of diabetes, history of cholesterol, and abdominal obesity).

**Fig 1:**
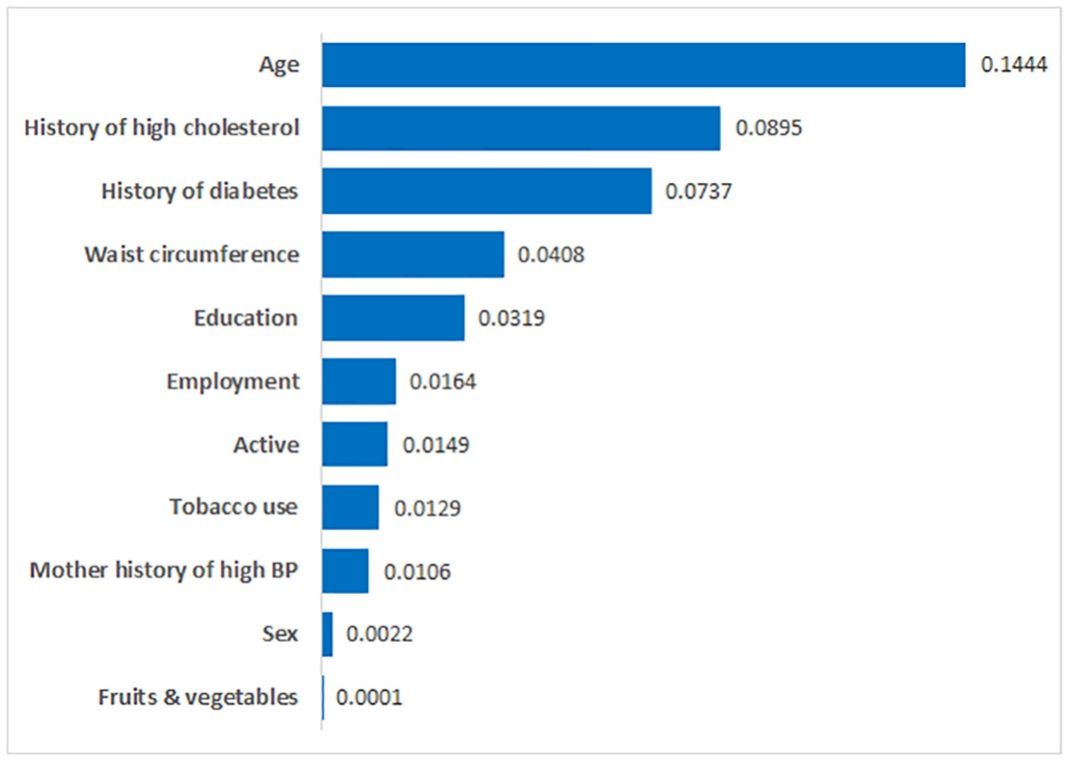
Relative importance of predictors of hypertension.

Fig 1 illustrates the order of the variables in terms of information gain in predicting hypertension. Age contributed the most to the prediction of hypertension with a contribution value of 0.144 followed by a lower contribution by the history of high cholesterol, history of diabetes and waist circumference (0.0895, 0.0737 and 0.0408, respectively). In this study, sex and fruits and vegetables had the least contribution value of 0.0022, and 0.0001 respectively, indicating minimal importance to the prediction of the disease.

### Performance of ML algorithms

**Fig 2:**
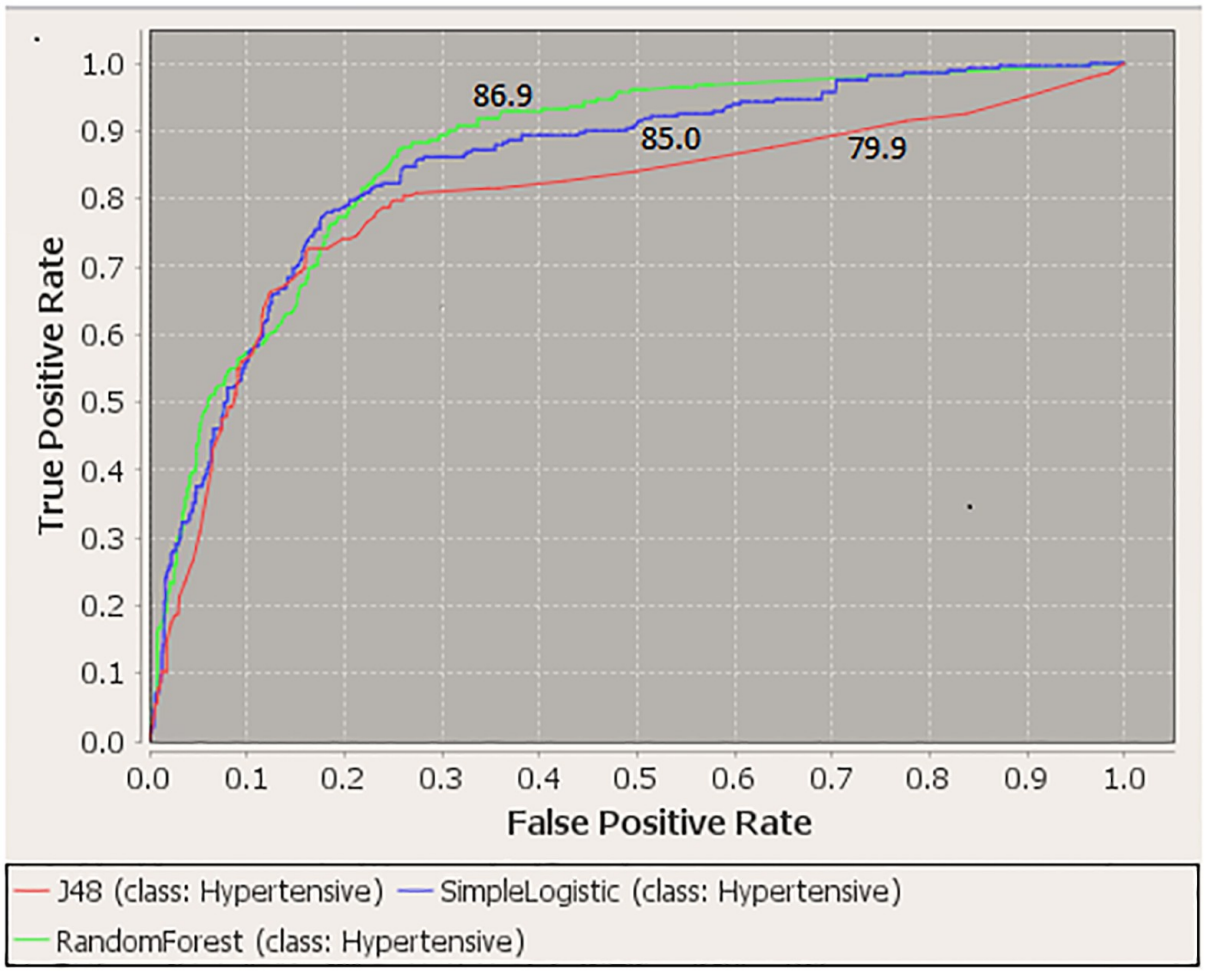
ROC curves for logistic regression, random forest and decision tree models.

Data was augmented before applying the algorithms; the total sample size increased to 1,128 with 282 hypertensive events. Table 2 represents the performance of the classifiers used and shows that random forest and decision tree exhibited more or less similar results, which were slightly better than those from the logistic regression in terms of accuracy, PPV, sensitivity and F-Measure. These measures for random forest were: accuracy = 82.1%, PPV = 81.4%, sensitivity = of 82.1% and F-measure = 81.6%. In terms of AUC, compared to logistic regression (85.0), random forest (86.9) performed similarly, and decision tree (79.9) had a significantly lower discrimination ability (p-value<0.05) (Fig 2). The training and test accuracy for the three algorithms were as follows, respectively: Logistic regression 81.9% and 81.9%; decision tree 86.0% and 82.1% and random forest 91.3% and 82.1%. These results indicate some overfitting when applying the random forest classifier.

**Table 2:** Performance of the machine learning algorithms.

Model	Accuracy	PPV	Sensitivity	F-Measure
**Logistic regression**	81.1%	80.1%	81.1%	80.3%
**Decision tree**	82.1%	81.2%	82.1%	81.4%
**Random Forest**	82.1%	81.4%	82.1%	81.6%

### Predicting hypertension

The algorithms may be used to make predictions of hypertension for a person using a new set of predictor values. Applying these values to the logistic regression model, for example, will produce a probability quantifying the likelihood that the person has a high risk of having hypertension. Using a cutoff, e.g., 0.5, this person may be categorized as potentially having hypertension if his probability was 0.5 or higher. Using the full training dataset and the Weka *SimpleLogistic* classifier with built-in variable selection, the resulting logit probability of hypertension is as follows:

$$\begin{array}{l} Logit(hypertension)=\;41\;+Younger\;than\;50\;*\;-0.86\;+\;Male\;*\;0.44\;+\\ \;Employed*\;-0.11\;+\;Tobacco\;use*\;-0.48\;+\;Active\;*\\ \;-0.1\;+\;Fruits\;and\;vegitables*\;-0.36\;+\\ \;Higher\;waist\;circumfrance\;*\;0.22\;+\;Diabetes\;*\;0.35\;+\\ \;Cholestrol-0.52\;+\;Mother\;history\;of\;high\;of\;BP*\;-0.30 \end{array}$$ (5)

**Fig 3:**
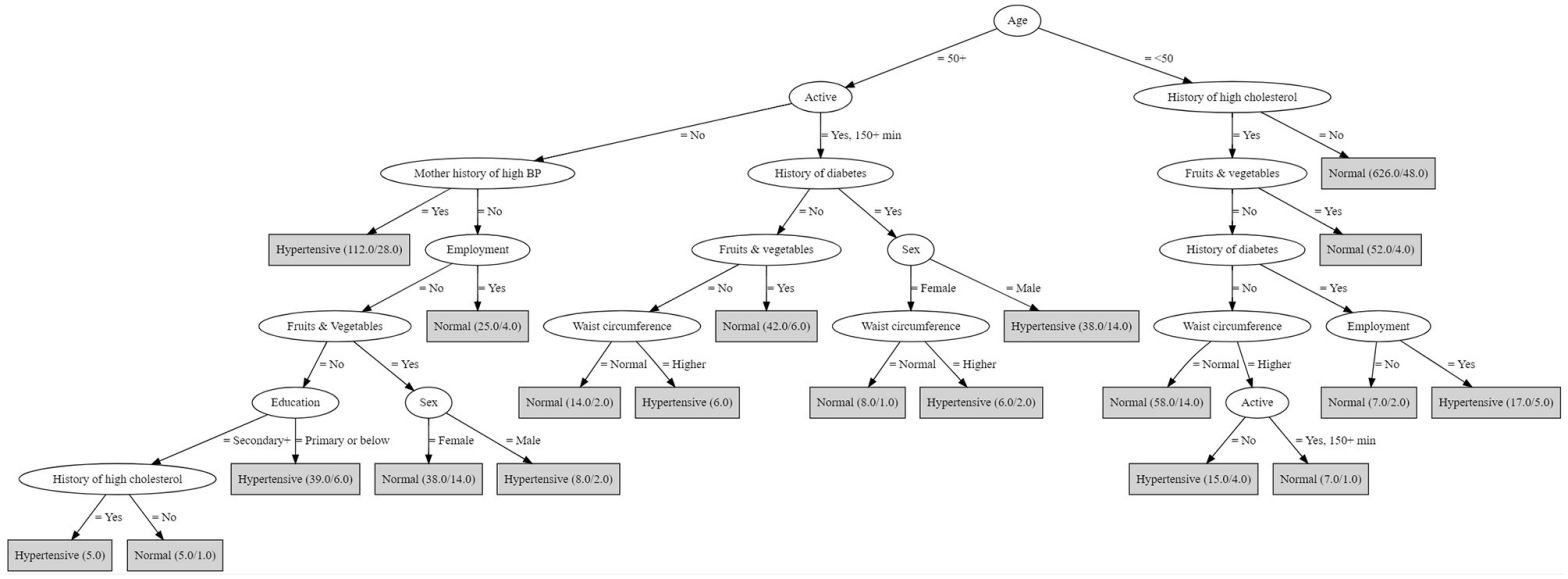
Decision tree.

Due to their popularity in ML and ease of use, decision trees are often used in many clinical setups. Fig 3 presents the visualization of the decision tree from this study. It consists of 8 levels, 20 leaf nodes, 18 internal/ decision nodes, and a root node. The root note is the most significant variable which has more information. The internal/ decision nodes represent the predictor variables that contributed the most to the prediction of hypertension based on best information gain; leaf nodes represent our outcome (hypertensive or normotensive). Variables are placed in branches according to their importance; in this study, age is the most important predictor.

If a person is aged more than 50 years, active, has no history of diabetes and consumes an adequate amount of fruits and vegetables, he/she is classified as being normotensive. This is an example of an interpretation from Fig 3. However, following a similar path but not consuming an adequate amount of fruits and vegetables, categorizing a person as hypertensive or not, depends on the waist circumference. If the person has a higher waist circumference and is not active, he/she is predicted to be hypertensive. Similarly, other paths are identified in Table 3. The first number in the brackets indicates the total number of instances reaching the leaf, while the second number indicates instances that were misclassified.

**Table 3:** The 20 rules extracted from the decision tree.

Twenty if-then rules extracted from the decision tree in Fig 3	
Rule 1	IF age is = 50+, not active, with a mother history of high BP, THEN a patient is hypertensive (112.0/28.0).
Rule 2	IF age is = 50+, not active, without a mother history of high BP, employed, THEN a patient is normal (25.0/4.0).
Rule 3	IF age is = 50+, not active, without mother history of high BP, not employed, not eating fruits & vegetables, with primary level of education or below, THEN a patient is hypertensive (39.0/6.0).
Rule 4	IF age is = 50+, not active, without mother history of high BP, not employed, not eating fruits & vegetables, with a secondary level of education or higher, with a history of high cholesterol, THEN a patient is hypertensive (5.0).
Rule 5	IF age is = 50+, not active, without mother history of high BP, not employed, not eating fruits & vegetables, with a secondary level of education or higher, without a history of high cholesterol, THEN a patient is normal (5.0/1.0).
Rule 6	IF age is = 50+, not active, without mother history of high BP, not employed, eating fruits & vegetables, a female, THEN a patient is normal (38.0/14.0).
Rule 7	IF age is = 50+, not active, without mother history of high BP, not employed, eating fruits & vegetables, a male, THEN a patient is hypertensive (8.0/2.0).
Rule 8	IF age is = 50+, active = 150+ min, without a history diabetes, eating fruits & vegetables, THEN a patient is normal (42.0/6.0).
Rule 9	IF age is = 50+, active = 150+ minutes, without a history diabetes, not eating fruits & vegetables, with normal waist circumference, THEN a patient is normal (14.0/2.0).
Rule 10	IF age is = 50+, active = 150+ minutes, without a history diabetes, not eating fruits & vegetables, with higher waist circumference, THEN a patient is hypertensive (6.0).
Rule 11	IF age is = 50+, active = 150+ minutes, with a history diabetes, a female, with normal waist circumference, THEN a patient is normal (8.0/1).
Rule 12	IF age is = 50+, active = 150+ minutes, with a history diabetes, a female, with higher waist circumference, THEN a patient is hypertensive (6.0/2.0).
Rule 13	IF age is = 50+, active = 150+ minutes, with a history diabetes, a male, THEN a patient is hypertensive (38.0/14.0).
Rule 14	IF age is = < 50, without a history of high cholesterol, THEN a patient is normal (626.0/48.0).
Rule 15	IF age is = < 50, with a history of high cholesterol, not eating fruits & vegetables, without a history of diabetes, with normal waist circumference, THEN a patient is normal (58.0/14.0).
Rule 16	IF age is = < 50, with a history of high cholesterol, not eating fruits & vegetables, without a history of diabetes, with higher waist circumference, not active, THEN a patient is hypertensive (15.0/4.0).
Rule 17	IF age is = < 50, with a history of high cholesterol, not eating fruits & vegetables, without a history of diabetes, with higher waist circumference, active = 150+ minutes, THEN a patient is normal (7.0/1.0).
Rule 18	IF age is = < 50, with a history of high cholesterol, eating fruits & vegetables, THEN a patient is normal (52.0/4.0).
Rule 19	IF age is = < 50, with a history of high cholesterol, not eating fruits & vegetables, with a history of diabetes, not employed, THEN a patient is normal (7.0/2.0).
Rule 20	IF age is = < 50, with a history of high cholesterol, not eating fruits & vegetables, with a history of diabetes, employed, THEN a patient is hypertensive (17.0/5.0).

## Discussion

As healthcare expenditures increase on chronic diseases with the increase in population, the significance of the application of this study in the public health field cannot be underestimated as the main goal of the predictive model is to predict the occurrence of hypertension. Consequently, such advancements facilitate forecasting trends. Implications of this study make it easier for the health workforce to identify individuals at high risk and those eligible for screening. Gaining such information on predicting hypertension will not only result in being advantageous to the individual by preventing adverse complications but also to the departmental and organizational levels decision-makers as it can facilitate in strategic decision making on certain areas of the healthcare system. Health professionals and policymakers can utilize the decision tree as a guide in establishing new programs by intervening or undergoing some corrective actions on existing ones and in understanding the most efficient method of resource allocation according to the number of individuals at risk in a region or per healthcare organization. In this research, models were developed to predict the occurrence of hypertension based on the presence of several predictors. This study assessed the ability of three ML algorithms to make these predictions based on non-intrusive baseline information. Age, gender, education level, employment, tobacco use, physical activity, adequate consumption of fruits and vegetables, abdominal obesity, history of diabetes, history of high cholesterol, and mother’s history high blood pressure were important predictors of hypertension. Algorithms generally had good prediction accuracy with random forest having better discrimination ability outperforming logistic regression and decision tree algorithms.

Predictive models using ML can generate robust diagnostic parameters as they produce accurate predictions using relationships between data, which verify its incremental validity [24]. For example, predictions of hypertension using conventional logistic regression are not further validated. However, when using ML, predictive logistic regression predicts the risk of hypertension by "learning" from data and validating the prediction. Furthermore, ML can substantiate which variable or set of variables is the most pragmatic in predicting hypertension. For expertise in this field, ML used in predictive models enables them to analyze and interpret clinical parameters identified as predictors for hypertension or not, along with other variables like daily lifestyle and other biological indicators [51]. Predictive models are constantly customized as prevention strategies as they are developed to tailor the intensity of the preventive measures to those at high risk of developing hypertension [16]. Moreover, they help in risk communication as they facilitate reaching out for those at high risk of developing hypertension and selecting them for clinical intervention, and lastly, allocate resources for future hypertension burdens.

Similar to our study, the medical literature identified certain biometric data such as age, obesity, and physical activity to be used in predicting hypertension [24, 44]. Consistent with other studies, our results revealed that older people are more vulnerable to develop the disease. One study found that older people are at a 2.7 times higher risk than younger ones to develop hypertension. This is manifested through the mechanism of the stiffening of the aorta and artery walls due to increased pulsatile stress that accelerates elastin degradation [5]. Longevity interventions and lifestyle factors are expected to improve cardiovascular fitness among the elderly [52]. Other studies reported that participants with metabolic syndrome have a higher likelihood of developing hypertension; diabetic participants and those with a history of high cholesterol are three times more likely to have hypertension compared with those who are not [53]. These results are concordant with our study.

Validated clinical studies identified the correlation between waist circumference and not having normal blood pressure [54, 55]; our findings are in agreement with these studies. Participants with higher waist circumference have a greater likelihood of developing hypertension by almost ten times than participants with normal waist circumference. This is due to the central accumulation of adipose tissues that are associated with elevated levels of triglycerides and uric acid, which are essential components of high blood pressure along with other components related to metabolic syndrome, such as insulin resistance [55].

Our study found that hypertension was more prevalent in males than females; this is similar to other studies [56]. According to a study conducted by Zekewos in 2019, the possible reason behind the gender disparity in hypertension prevalence is due to biological distinction and behavioral risk factors like physical activity, alcohol consumption, or smoking [6]. Being a woman with less addiction to smoking and alcohol than men is considered protective. Furthermore, women are known to be more involved and investing in the utilization of healthcare services regularly, which makes them more likely to achieve better health outcomes than men [6]. However, in this study, although hypertension was more prevalent in males than females, the difference was not statistically significant. This might be due to the unique demographic composition in Qatar. The labor force, which is mostly males, depart the country before retiring at the age of 60 years. As people develop hypertension in older ages, the country remains with younger males and, therefore with a lower count of males with hypertension.

Other hypertension risk factors like alcohol consumption, kidney disease, and salt consumption were not included in our study. Qatar is an Islamic country where consumption of alcohol is forbidden, and the sale of alcohol is limited due to the religious rulings [57]. Therefore, such data was not collected. Chronic kidney disease was also not evaluated because only 6 subjects in our sample suffered from this disease. Studies have shown that the reduction of salt consumption prevents hypertension [58]. We were not able to assess this association in this study due to data limitations.

In the current study, a "healthy volunteer" selection bias was present due to the underrepresentation of the study sample to the general population of Qatar. QBB participants are recruited either via social media, through the QBB website, or by personal recommendations of family and friends [30]. In our study, most participants were Qataris (85%); 12% were long term Arab residents, and 3% were long-term non-Arab residents [30]. On the contrary, only 15% of the general population are Qataris [59]; as such other characteristics like employment and history of diseases vary. Sample size, inclusion, and selection of participants will have an overall effect on the performance of the algorithms due to the common issue of underestimation that was faced in previous studies that have utilized ML. Although the sample is not representative of the population in Qatar, it would have been adequate for generalization of results and proper assessment of the performance of the algorithms if our study had a larger sample size [60, 61].

Logistic regression showed better discrimination ability than the decision tree in terms of predicting the risk of hypertension because minimal changes and variability in the data can create high instability in decision trees [62]. This was inconsistent with previous studies [63]. Discrepancies in classification models’ performance are related to several factors, including the differences in technologies, procedures, and assumptions that operate under each model, differences in the dataset characteristics, and the number of predictors used as well as the model building technique and sample size [64]. Similar to performance results found in some studies [65, 66], random forest performed better than the decision tree. This result is expected, as random forests with multiple single trees are known to be robust techniques than a single decision tree. Random forest considers the outputs of multiple decision trees, which addresses their sensitivity to the training data resulting in a reduction in the variance within the outcome of the data [47]. In our study, logistic regression and random forest were comparable in their discrimination ability. This result is consistent with other simulation studies with sample sizes of less than 1,000 observations [67]. Random forest will outperform logistic regression when there are nonlinearities and interactions among predictors. It is potential that in our hypertension prediction model, predictors related additively in a linear fashion similar to other clinical predictor models, thus defying the superiority of random forest [68, 69].

This study provides a foundation for the prediction of hypertension depending on a number of risk factors. Most of the predictor variables used in this study were in line with other published data [6, 23, 51]. The strength of this study is the focus on non-invasive data and exploring more than one algorithm to predict hypertension.

Nonetheless, this study has some limitations. First, the prediction algorithms used in this study were applied to relatively small sample size. The augmented data in our study had a sample size of 1,128 with 282 hypertensive events. Using five-fold cross-validation means that the observations used to train the models amount to 902 (= 1128*0.8) with 225 (= 902*282/1128) hypertensive events. For logistic regression, the minimum training data size is determined by the number of events rather than the number of observations with 10 events per variable as a general rule of thumb [70]. Since we have 11 variables in our logistic model, the minimal number of events is 10*11 = 110, which is beyond our 225 number of events. In general, the algorithm used in this study performs better using larger sample sizes [63], particularly those based on decision trees. Compared to logistic regression, these algorithms may need 10 times as many events for each predictor to achieve a small amount of overfitting [71]. Thus results of this study should be interpreted with caution.

Second, our results were from a single, cross-sectional sample to predict hypertension using a number of predictors, and these predictors may change over time; any causal associations between predictors and hypertension is thus limited. Finally, the study population did not represent the population of Qatar in terms of age, nationality, and other characteristics. The QBB study, from which the data of this study was extracted, collects records from healthy volunteers, which introduced selection bias, therefore, limiting the generalizability of the results external to the QBB population, particularly with our limited sample size.

## Conclusion

ML was utilized to generate a decision tree that was helpful in the prediction of hypertension in data obtained from QBB without using non-invasive procedures. Using predictive models to identify potential hypertensive people have several real-world implications, including tailoring preventive solutions to those at high risk of developing hypertension. Through accurate risk communication, predictive models can help in the improvement of shared health decision making concerning people at higher risk of developing the disease. Predictive models for hypertension can also help in deciding on the level of interventions needed within the community and thus assuring a positive impact. Future research should consider improving the predictive accuracy of models by using the algorithms in larger general populations to avoid the healthy population effect. This research may be extended by assessing other predictors and using different prediction algorithms like artificial neural network, support vector machine, naive bayes classifier, and gradient boosting machines.
